# Supplementary material for: Sociodemographic and educational factors associated with mental health disorders in medical students of clinical years: A multicenter study in Peru
Source: PLoS One. 2023 Jun 26;18(6):e0286338. doi: 10.1371/journal.pone.0286338 (PMC10292711; doi:10.1371/journal.pone.0286338)
Supplement: S1 Dataset — (DOCX) [file pone.0286338.s001.docx]

**S1 Dataset. (XLSX) (*Database)***

[10.6084/m9.figshare.21874950](https://doi.org/10.6084/m9.figshare.21874950)
