# Supplementary material for: Sociodemographic and educational factors associated with mental health disorders in medical students of clinical years: A multicenter study in Peru
Source: PLoS One. 2023 Jun 26;18(6):e0286338. doi: 10.1371/journal.pone.0286338 (PMC10292711; doi:10.1371/journal.pone.0286338)
Supplement: S2 Dataset — (DOCX) [file pone.0286338.s002.docx]

**S2 Dataset. (XLSX) (*Commands used for statistical analysis)***

[10.6084/m9.figshare.21874965](https://doi.org/10.6084/m9.figshare.21874965)

*Análisis AE_SM JPEZV

save ae_sm03.dta

*Análisis exploratorio

--------------------------------------------------------------

*Tabla1 - Análisis univariado

// Variables categóricas//

tab sexo

label define sexo 0 "Femenino" 1 "Masculino"

label values sexo sexo

tab sexo

tab religion

gen religion_catego=religion

recode religion_catego (0=1) (1=2) (2=3) (3/12=4)

label define religion_catego 1 "Adventista" 2 "Ateo" 3 "Católico/a" 4 "Otros"

label values religion_catego religion_catego

tab religion_catego

gen religion_categori=religion_catego

recode religion_categori (2=1) (3=2) (1/4=3)

label define religion_categori 1 "Ateo" 2 "Católico/a" 3 "Otros"

label values religion_categori religion_categori

tab religion_categori

tab estadocivil

gen estadocivil_cate=estadocivil

recode estadocivil_cate (0=1) (1/6=2)

label define estadocivil_cate 1 "Soltero" 2 "Comp/Cas/Nov/Otros"

label values estadocivil_cate estadocivil_cate

tab estadocivil_cate

tab dieta

gen dieta_cat=dieta

recode dieta_cat (0=1) (1/5=2)

label define dieta_cat 1 "No vegetariana" 2 "Variedades/Otras"

label values dieta_cat dieta_cat

tab dieta_cat

tab lugar_vive

gen lugar_vive_cat=lugar_vive

recode lugar_vive_cat (0=1) (1/4=2)

label define lugar_vive_cat 1 "Casa" 2 "Depa/Resid univ/Pensión/Otros"

label values lugar_vive_cat lugar_vive_cat

tab lugar_vive_cat

tab internado

label define internado 0 "No" 1 "Si"

label values internado internado

tab internado

tab colegio

label define colegio 0 "No" 1 "Si"

label values colegio colegio

tab colegio

tab instrucción_jefe

gen instrucción_jefe_cat=instrucción_jefe

recode instrucción_jefe_cat (0/1=1) (2/4=2)

label define instrucción_jefe_cat 1 "Estudios prim/sec" 2 "Estudios univ/tec/posgrado"

label values instrucción_jefe_cat instrucción_jefe_cat

tab instrucción_jefe_cat

tab carga_academica

label define carga_academica 0 "No" 1 "Si"

label values carga_academica carga_academica

tab carga_academica

tab otracarrera

label define otracarrera 0 "No" 1 "Si"

label values otracarrera otracarrera

tab otracarrera

tab beca

label define beca 0 "No" 1 "Si"

label values beca beca

tab beca

tab trabajo

label define trabajo 0 "No" 1 "Si"

label values trabajo trabajo

tab trabajo

tab necesidad_trab

label define necesidad_trab 0 "No" 1 "Si"

label values necesidad_trab necesidad_trab

tab necesidad_trab

tab ant_enfermedad

label define ant_enfermedad 0 "No" 1 "Si"

label values ant_enfermedad ant_enfermedad

tab ant_enfermedad

tab continua_enf

label define continua_enf 0 "No" 1 "Si"

label values continua_enf continua_enf

tab continua_enf

tab dxcovid19

label define dxcovid19 0 "No" 1 "Si"

label values dxcovid19 dxcovid19

tab dxcovid19

******Establezcacuálessonlasraz

tab razon1

label define razon1 0 "No" 1 "Si"

label values razon1 razon1

tab razon1

label define razon2 0 "No" 1 "Si"

label values razon2 razon2

tab razon2

label define razon3 0 "No" 1 "Si"

label values razon3 razon3

tab razon3

label define razon4 0 "No" 1 "Si"

label values razon4 razon4

tab razon4

label define razon5 0 "No" 1 "Si"

label values razon5 razon5

tab razon5

label define razon6 0 "No" 1 "Si"

label values razon6 razon6

tab razon6

label define razon7 0 "No" 1 "Si"

label values razon7 razon7

tab razon7

tab semestre

label define semestre 4 "5to ciclo" 5 "6to ciclo" 6 "7mo ciclo" 7 "8vo ciclo" 8 "9no ciclo" 9 "10mo ciclo" 10 "11vo ciclo" 11 "12vo ciclo" 12 "13vo ciclo" 13 "14vo ciclo"

label values semestre semestre

tab semestre

numlabel, add

tab semestre

gen semestre_cat=semestre

recode semestre_cat (4/5=1) (6/7=2) (8/9=3) (10/11=4) (12/13=5)

label define semestre_cat 1 "3er año" 2 "4to año" 3 "5to año" 4 "6to año" 5 "7mo año"

label values semestre_cat semestre_cat

tab semestre_cat

tab regio_css

label define regio_css 1 "Costa" 2 "Sierra" 3 "Selva"

label values regio_css regio_css

tab regio_css

tab tipodeuniv

label define tipodeuniv 1 "Privada" 2 "Nacional"

label values tipodeuniv tipodeuniv

tab tipodeuniv

//Instrumentos//

*AE

gen amb_educ =(ae_1+ae_2+ae_3+ae_4+ae_5+ae_6+ae_7+ae_8+ae_9+ae_10+ae_11+ae_12+ae_13+ae_14+ae_15+ae_16+ae_17+ae_18+ae_19+ae_20+ae_21+ae_22+ae_23+ae_24+ae_25+ae_26+ae_27+ae_28+ae_29+ae_30+ae_31+ae_32+ae_33+ae_34+ae_35+ae_36+ae_37+ae_38+ae_39+ae_40+ae_41+ae_42+ae_43+ae_44+ae_45+ae_46+ae_47+ae_48+ae_49+ae_50)

tab amb_educ

gen amb_educ_catego=amb_educ

recode amb_educ_catego (0/50=1) (51/100=2) (101/150=3) (151/200=4)

label define amb_educ_catego 1 "AE muy pobre" 2 "AE con muhcos problemas" 3 "AE mas positivo que negativo" 4 "AE excelente"

label values amb_educ_catego amb_educ_catego

tab amb_educ_catego

tab amb_educ_cat

numlabel, add

tab amb_educ_catego

gen amb_educ_categor=amb_educ_catego

recode amb_educ_categor (1/2=2) (3/4=1)

label define amb_educ_categor 1 "AE bueno" 2 "AE malo"

label values amb_educ_categor amb_educ_categor

tab amb_educ_categor

*ANSIEDAD

gen ansiedad_sum=(ansiedad_1+ansiedad_2+ansiedad_3+ansiedad_4+ansiedad_5+ansiedad_6+ansiedad_7)

tab ansiedad_sum

gen ansiedad_sum_cat=ansiedad_sum

recode ansiedad_sum_cat (0/4=1) (5/9=2) (10/14=3) (15/21=4)

label define ansiedad_sum_cat 1 "Normal" 2 "Ligera" 3 "Moderada" 4 "Severa"

label values ansiedad_sum_cat ansiedad_sum_cat

tab ansiedad_sum_cat

gen ansiedad_sum_categ=ansiedad_sum_cat

recode ansiedad_sum_categ (1=1) (2/4=2)

label define ansiedad_sum_categ 1 "No ansiedad" 2 "Si ansiedad"

label values ansiedad_sum_categ ansiedad_sum_categ

tab ansiedad_sum_categ

*DEPRESIÓN

gen depresion_sum=(depresion_1+depresion_2+depresion_3+depresion_4+depresion_5+depresion_6+depresion_7+depresion_8+depresion_9)

tab depresion_sum

gen depresion_sum_cat=depresion_sum

recode depresion_sum_cat (0/4=1) (5/9=2) (10/14=3) (15/19=4) (20/27=5)

label define depresion_sum_cat 1 "Normal" 2 "Leve" 3 "Moderada" 4 "Moderadamente severo" 5 "Severo"

label values depresion_sum_cat depresion_sum_cat

tab depresion_sum_cat

gen depresion_sum_categ=depresion_sum_cat

recode depresion_sum_categ (1=1) (2/5=2)

label define depresion_sum_categ 1 "No depresión" 2 "Si depresión"

label values depresion_sum_categ depresion_sum_categ

tab depresion_sum_categ

/Variables numericas/

tabstat edad, s(p50 p25 p75 mean sd skewness kurtosis)

tabstat horas_semana, s(p50 p25 p75 mean sd skewness kurtosis)

tabstat habitaciones, s(p50 p25 p75 mean sd skewness kurtosis)

tabstat horas_dia, s(p50 p25 p75 mean sd skewness kurtosis)

tabstat personas_vive, s(p50 p25 p75 mean sd skewness kurtosis)

---------------------------------------------------------------

*Tabla2 - Análisis bivariado

Ansiedad

tab sexo ansiedad_sum_categ, row chi2

tab estadocivil_cate ansiedad_sum_categ, row chi2

tab religion_categori ansiedad_sum_categ, row chi2

tab dieta_cat ansiedad_sum_categ, row chi2

tab lugar_vive_cat ansiedad_sum_categ, row chi2

tab internado ansiedad_sum_categ, row chi2

tab semestre_cat ansiedad_sum_categ, row chi2

tab carga_academica ansiedad_sum_categ, row chi2

tab beca ansiedad_sum_categ, row chi2

tab ant_enfermedad ansiedad_sum_categ, row chi2

tab dxcovid19 ansiedad_sum_categ, row chi2

tab regio_css ansiedad_sum_categ, row chi2

tab tipodeuniv ansiedad_sum_categ, row chi2

tab razon1 ansiedad_sum_categ, row chi2

tab razon2 ansiedad_sum_categ, row chi2

tab razon3 ansiedad_sum_categ, row chi2

tab razon4 ansiedad_sum_categ, row chi2

tab razon5 ansiedad_sum_categ, row chi2

tab razon6 ansiedad_sum_categ, row chi2

tab razon7 ansiedad_sum_categ, row chi2

tab amb_educ_categor ansiedad_sum_categ, row chi2

kwallis personas_vive, by(ansiedad_sum_categ)

kwallis habitaciones, by(ansiedad_sum_categ)

kwallis horas_dia, by(ansiedad_sum_categ)

kwallis horas_semana, by(ansiedad_sum_categ)

-----------------------------------------

Depresion

tab sexo depresion_sum_categ, row chi2

tab estadocivil_cate depresion_sum_categ, row chi2

tab religion_categori depresion_sum_categ, row chi2

tab dieta_cat depresion_sum_categ, row chi2

tab lugar_vive_cat depresion_sum_categ, row chi2

tab internado depresion_sum_categ, row chi2

tab semestre_cat depresion_sum_categ, row chi2

tab carga_academica depresion_sum_categ, row chi2

tab beca depresion_sum_categ, row chi2

tab ant_enfermedad depresion_sum_categ, row chi2

tab dxcovid19 depresion_sum_categ, row chi2

tab regio_css depresion_sum_categ, row chi2

tab tipodeuniv depresion_sum_categ, row chi2

tab razon1 depresion_sum_categ, row chi2

tab razon2 depresion_sum_categ, row chi2

tab razon3 depresion_sum_categ, row chi2

tab razon4 depresion_sum_categ, row chi2

tab razon5 depresion_sum_categ, row chi2

tab razon6 depresion_sum_categ, row chi2

tab razon7 depresion_sum_categ, row chi2

tab amb_educ_categor depresion_sum_categ, row chi2

kwallis personas_vive, by(depresion_sum_categ)

kwallis habitaciones, by(depresion_sum_categ)

kwallis horas_dia, by(depresion_sum_categ)

kwallis horas_semana, by(depresion_sum_categ)

---------------------------------------------------------------

*Tabla 3 - Análisis multivariado

*Regresión simple

glm ansiedad_sum_categ i.sexo i.ant_enfermedad i.dxcovid19 i.regio_css i.carga_academica razon1 razon2 razon3 razon4 razon5 razon6 razon7 i.tipodeuniv i.amb_educ_categor, fam(poisson) link(log) eform robust cluster(tipodeuniv)

glm depresion_sum_categ i.sexo i.ant_enfermedad i.tipodeuniv i.amb_educ_categor, fam(poisson) link(log) eform robust cluster(tipodeuniv)

*Regresión múltiple//AE-SM

glm ansiedad_sum_categ i.sexo i.ant_enfermedad i.dxcovid19 i.regio_css i.carga_academica i.tipodeuniv i.amb_educ_categor,fam(poisson) link(log) eform robust cluster(tipodeuniv)

glm depresion_sum_categ i.sexo i.ant_enfermedad i.tipodeuniv i.amb_educ_categor, fam(poisson) link(log) eform robust cluster(tipodeuniv)
