# Supplementary material for: Sociodemographic and educational factors associated with mental health disorders in medical students of clinical years: A multicenter study in Peru
Source: PLoS One. 2023 Jun 26;18(6):e0286338. doi: 10.1371/journal.pone.0286338 (PMC10292711; doi:10.1371/journal.pone.0286338)
Supplement: Attachment — Submitted filename: PONE - 22 - 28915 Review.docx [file pone.0286338.s003.docx]

Thank you for inviting me to review this article. It is well written and has a high potential for publication. However, please find my comments below to improve the manuscript.

Title –

- It is ok but the word ‘multicenter’ and ’24 Peruvian universities’ might be repetitive. Therefore, the methodology of the study might replace and be additionally mentioned in the title.

Abstract

- Abbreviations are suggested to be clarified.
- The reported statistics should follow the journal’s format.
- The last sentence of the result might be considered as the discussion. Meanwhile, the conclusion could be shorter and clearly sum up the finding, discussion, and further suggestion.

Introduction

- The sequence of each paragraph might be rearranged to make the section easier for readers. For example, mental morbidities might be stated following the importance of EE which is directedly affected by the pandemic as the authors firstly mentioned.
- After that, mental illness among medical students should be focused. There are several previous studies in respect to such study population, including

(1) Zeng W, Chen R, Wang X, et al. Prevalence of mental health problems among medical students in China: A meta-analysis. *Medicine (Baltimore)*. 2019;98(18):e15337. doi:10.1097/MD.0000000000015337
(2) Chiddaycha M, Wainipitapong S. Mental health among Thai medical students: Preadmission evaluation and service utilization. Health Sci Rep. 2021 Oct 9;4(4):e416. doi: 10.1002/hsr2.416. PMID: 34646944; PMCID: PMC8501376.
(3) Pham T, Bui L, Nguyen A, et al. The prevalence of depression and associated risk factors among medical students: An untold story in Vietnam. *PLoS One*. 2019;14(8):e0221432. doi:10.1371/journal.pone.0221432
(4) Puthran R, Zhang MWB, Tam WW, et al. Prevalence of depression amongst medical students: a meta-analysis. *Med Educ*. 2016;50(4):456-468. doi:10.1111/medu.12962
(5) Pacheco JP, Giacomin HT, Tam WW, Ribeiro TB, Arab C, Bezerra IM, Pinasco GC. Mental health problems among medical students in Brazil: a systematic review and meta-analysis. Braz J Psychiatry. 2017 Oct-Dec;39(4):369-378. doi: 10.1590/1516-4446-2017-2223. Epub 2017 Aug 31. PMID: 28876408; PMCID: PMC7111407.

. . and so on.

- Moreover, consequences of those mental conditions toward medical students could be stated (i.e., dropout - doi: 10.1186/s12909-022-03527-z / suicide - doi: 10.1097/ACM.0000000000002507 / and many more) to emphasize and justify your study of linkage between mental health and EE. Finally, the last paragraph then acclaimed your study justification and the objective of the study.
- Though I am not a native English user, I believe that the manuscript will gain benefit from English editing.

Methods –

- The authors should clarify whether they select clinical year students as a sample, but the title referred to any medical students.
- Page 4 Line 108-110 – Is it a typo?
- Page 5 Line 111-119 – The COVID-19 situation affecting medical education in Peru should be stated in the introduction instead of the method section.
- Reference of sample size calculation is recommended to be added.
- The snowball sampling is quite confusing. Please kindly explain more about this, because authors later stated about advertising the surveys through social network platforms.
- Inclusion and exclusion criteria should be clearly stated. Also, the timeline of the study should be mentioned for the surveillance timepoint is vital for mental health screening according to the nature of academic/examination effect throughout the year, and the data of each study year would be a better representative if they were collected at the same timepoint.
- I think some details are redundant (i.e., the process in the Google Forms) and could be shorten or removed.
- Some items measured by each questionnaire should be exemplified for readers might easily view the scopes of each test, especially the EE which might not be familiar in readers from the field of psychiatry.
- What is ES (Page 7 Line 179)?
- Statistical analysis subheading should provide general statistical usage of the manuscript and not link with the finding. I am not sure about the median and IQR used in terms of age because it is not normally-distributed. Thus, I recommend more general statement such as ‘either Mean +/- SD or Median (IQR) was used regarding the distribution of data, normally and not-normally distributed, respectively’.
- Please kindly check terms ‘Multivariate’ and ‘Multiple’ analysis. Also, I am not sure whether authors wanted to infer ‘Bivariate’ in Page 7 Line 189, or not.

Results –

- Please kindly check the reported age, is it a mean with IQR?
- What is the difference between Half of them (56.4%) and More than half (58.0%) in Page 8 Line 204-205?
- Please kindly recheck the spelling (and meaning) in the table. For example, ‘Caracteristicas’ or ‘Typo of property’.
- Also, please kindly recheck the reported percent. For example in the marital status, if single is accountable for 81.9%, the reset should be 146/808 x 100 = 18.06 >> 18.1).
- Some definitions were unclear and needed explanation (i.e., human medicine or full academic load).
- Authors stated about bivariate analysis in Table 2 (Page 10 Line 221-222) which was not consistent with the declaration below the table mentioning about the chi-square test. If the bivariate analysis was done, I suggest to add an additional table with proper statistical details including OR, 95% CI, or P-value. Also, some of the variables might need the Fischer exact test. Please kindly reconfirm with statistical experts with all statistical analysis.
- I am not a statistician and uncertain about the term ‘Simple and multiple regression’ in multivariate analysis. Thus, I cannot give many comments about this issue. Also, please recheck the term used with the abstract either.
- Was P-value of being male and anxiety significant with the P-value 0.010?
- The Figure is not necessary. However, authors might elaborate some comparative variables including those with and without mental morbidities in the figure.

Discussion –

- The authors acclaimed that the finding was superior to previous studies (Page 15 Line 237). Please kindly provide evidence for the statement.
- The citation number 14 cannot be used to report more frequent depression in male students.
- Specifically, authors are recommended to discuss in the context of the clinical medical students during the COVID-19, which is unique and worth discussing.
- More strengths of this study should be mentioned.
- Limitations should include the generalizability for either sociocultural contexts, pandemic situations, or the clinical year only that recruited in the study.

Reference –

Please recheck the format; for example number 42,

*Yt N, T EM, S W, D J, Cm W, Ha H. The association between COVID-19*

*546 diagnosis or having symptoms and anxiety among Canadians: A repeated cross547*

*sectional study. Anxiety, stress, and coping. 2021;34(5).*

*548 doi:10.1080/10615806.2021.1932837*
